# An outbreak of locally acquired *Plasmodium vivax* malaria among migrant workers in Oman

**DOI:** 10.1051/parasite/2017028

**Published:** 2017-07-11

**Authors:** Bruno Simon, Fatimata Sow, Said K. Al Mukhaini, Seif Al-Abri, Osama A.M. Ali, Guillaume Bonnot, Anne-Lise Bienvenu, Eskild Petersen, Stéphane Picot

**Affiliations:** 1 Malaria Research Unit, SMITh, ICBMS UMR 5246, University of Lyon, Campus Lyon-Tech La Doua 43 Boulevard du 11 Novembre 1918 69622 Villeurbanne France; 2 The Department of Malaria, Directorate General for Disease Surveillance and Control, Ministry of Health P. O. Box 393 Postal Code 113 Muscat Oman; 3 Directorate General for Disease Surveillance and Control, Ministry of Health P. O. Box 2657 CPO 111 Muscat Oman; 4 Service Pharmacie, Hospices Civils de Lyon 103 Grande Rue de la Croix-Rousse 69317 Lyon France; 5 Department of Infectious Diseases, The Royal Hospital P. O. Box 1331 CPO 111 Muscat Oman; 6 Institute of Clinical Medicine, Faculty of Health Sciences, University of Aarhus Palle Juul-Jensens Boulevard 82 8200 Aarhus N Denmark; 7 Institut de Parasitologie et Mycologie Médicale, Hospices Civils de Lyon 103 Grande Rue de la Croix-Rousse 69317 Lyon France

**Keywords:** Malaria, *Plasmodium vivax*, Outbreak, Oman, Genotyping, Temperate genotype

## Abstract

*Plasmodium vivax* is the most widely distributed human malaria parasite. Outside sub-Saharan Africa, the proportion of *P. vivax* malaria is rising. A major cause for concern is the re-emergence of *Plasmodium vivax* in malaria-free areas. Oman, situated in the south-eastern corner of the Arabian Peninsula, has long been an area of vivax malaria transmission but no locally acquired cases were reported in 2004. However, local transmission has been registered in small outbreaks since 2007. In this study, a local outbreak of 54 cases over 50 days in 2014 was analyzed retrospectively and stained blood slides have been obtained for parasite identification and genotyping. The aim of this study was to identify the geographical origin of these cases, in an attempt to differentiate between imported cases and local transmission. Using circumsporozoite protein (*csp*), merozoite surface protein 1 (*msp1*), and merozoite surface protein 3 (*msp3*) markers for genotyping of parasite DNA obtained by scrapping off the surface of smears, genetic diversity and phylogenetic analysis were performed. The study found that the samples had very low genetic diversity, a temperate genotype, and a high genetic distance, with most of the reference strains coming from endemic countries. We conclude that a small outbreak of imported malaria is not associated with re-emergence of malaria transmission in Oman, as no new cases have been seen since the outbreak ended.

## Abstract (fr)

*Plasmodium vivax* est le parasite du paludisme humain le plus répandu. En dehors de l’Afrique subsaharienne, la proportion de paludisme par *P. vivax* augmente. Une préoccupation majeure est la réémergence de *Plasmodium vivax* dans les zones sans paludisme. Oman, situé dans le coin sud-est de la péninsule arabique, est depuis longtemps une zone de transmission de *P. vivax* mais aucun cas localement acquis n’a été signalé en 2004. Cependant, la transmission locale a été rapportée dans de petites épidémies depuis 2007. Dans ce travail, une épidémie locale de 54 cas pendant 50 jours en 2014 a été analysée rétrospectivement et des lames de sang colorées ont été obtenues pour l’identification et le génotypage du parasite. L’objectif de cette étude était d’identifier l’origine géographique de ces cas, afin de différencier les cas importés et la transmission locale. Une analyse de la diversité génétique et une analyse phylogénétique ont été effectuées en utilisant les marqueurs *csp*, *msp1* et *msp3* pour le génotypage de l’ADN des parasites, obtenu en grattant la surface des frottis. L’étude a révélé que les échantillons avaient une très faible diversité génétique, un génotype tempéré et une grande distance génétique avec la plupart des souches de référence provenant de pays endémiques. Nous concluons qu’une petite flambée de paludisme importé n’est pas associée à la réémergence de la transmission du paludisme en Oman, car aucun nouveau cas n’a été observé après la fin de la flambée.

## Introduction

Malaria due to *P. vivax* results in considerable morbidity and mortality [2, 6, 11, 16]. *P. vivax* is currently the most widely distributed human malaria parasite with an estimated 2.5 billion people at risk [14]. *P. vivax* accounts for more than half of all malaria cases in Latin America, the Middle East, Asia, and the Western Pacific. Outside sub-Saharan Africa, the proportions of *P. vivax* malaria are rising, a clear indication of the resilience of this parasite to control measures [10, 40]. *P. vivax* has long been considered a neglected parasite while its socio-economic burden in endemic areas is huge. *P. vivax* exist in a temperate form with a long latency period up to nine months and a tropical form with a short latency period [4, 5, 20, 28, 39]. The two forms can be separated by haplotype analysis [24, 38].

The risk of introducing *P. vivax* into previously malaria-free areas is related to population movements [28], as recently demonstrated in Greece [1, 8, 34]. Thus, extensive knowledge about local epidemiology and the genetics of *P. vivax* malaria is of the highest importance in order to achieve effective control measures in malaria-endemic areas.

Oman is situated in the south-eastern corner of the Arabian Peninsula, bordering the Kingdom of Saudi Arabia, the United Arab Emirates, and Yemen. The summer is hot and humid and the winter is colder with some rain. The population in 2017 was estimated to be 2.5 million Omani nationals plus an expatriate population of 2.12 million (National Centre for Statistics and Information: https://www.ncsi.gov.om/Pages/NCSI.aspx). Oman has long been an area of *P. vivax* malaria transmission (33,000 cases in 1990), but control aiming at eradication was started in 1991 and in 2000 the annual parasite incidence had been reduced to 1 per 10,000 population [25]. Oman registered no locally transmitted cases of malaria cases in 2004 for the first time after the eradication program started. However, in September 2007, a focus of local transmission (four cases) was found in Dakhiliya governorate and in 2008 in North Batinah governorate (eight cases). Secondary cases occurred in North Sharqiya governorate in 2010, North Sharqiya and Dakhiliya in 2011, and North and South Sharqiya and North Batinah in 2012. Local transmission was again found in 2013 in Al Dakhiliya, North, Al Batinah, and South Sharqiya governorates [25]. The number of imported cases (99.24% of all cases) started to show a decrease from 2012 (2029 cases) to 2013 (1440 cases), while 11 locally acquired cases were detected in 2013. These locally acquired cases most probably reflect repeated re-introduction from the high number of migrant workers from the Indian subcontinent visiting their home countries where malaria, especially *P. vivax*, is endemic.

Several molecular markers have been tested to examine parasite population diversity [7]. Microsatellites are probably the most informative markers to conduct an outbreak analysis [3, 12, 22, 23]; however, this method requires sufficient amounts of good quality DNA, and is not suitable for a retrospective analysis restricted to samples of low quality and quantity obtained after scraping of Giemsa-stained blood smears. Among the other markers generally described, genes encoding the circumsporozoite protein (*csp*) [17], merozoite surface protein 1 (*msp1*), and merozoite surface protein 3 (*msp3*) have been used extensively [15, 17, 30, 32, 35]. These genetic polymorphisms have been published from samples collected in many endemic areas, leading to the possibility of investigating the origin of an outbreak by sequence comparisons. However, although a high number of sequences of these markers are available in databases, most of them have been collected in areas with high endemicity, including Brazil, India, and Papua New Guinea. Little is known about *Plasmodium vivax* parasites circulating in areas with low and very low endemicity or causing outbreaks in areas free of malaria.

This study is a retrospective analysis of DNA samples collected in Oman from Giemsa-stained blood films obtained from 45 patients diagnosed with malaria in 2014 and collected during a short period of less than 50 days in a focus area of 16 km^2^. It is important to determine whether this malaria transmission was introduced into Oman repeatedly from migrant workers coming from malaria-endemic areas, or was locally re-established.

## Material and methods

### Ethical clearance

This study did not impact the diagnosis, treatment, or follow-up of patients since samples were obtained after the end of the outbreak. Ethical clearance was obtained from the Ministry of Health, Sultanate of Oman.

### Malaria outbreak

#### Time of outbreak

The first case was registered on 22 September 2014 and the last case on 9 November 2014.

#### Geography

All cases were found within a 4 × 4 km area of Mabela, in the Seeb district of Muscat governorate, Oman. This is an area with intensive construction of new buildings.

#### Breeding sites

Environmental investigation was initiated and seven open water tanks related to building construction sites and 15 pools used for irrigation were sampled. Three water tanks and five pools were identified with *Anopheles* larvae. The malaria vector in Oman is *Anopheles culicifacies*.

#### Malaria patients

Fifty-four cases were registered from the area, 52 men and 2 women (mean age = 32 ± 8). All patients had *P. vivax* circulating blood stages as detected by experienced microscopists. One patient (Omani national) had recently traveled to Pakistan and was excluded from the study. The remaining 53 patients were all expatriates from India (*n* = 14), Pakistan (*n* = 6), Bangladesh (*n* = 32), and Egypt (*n* = 1). The epidemiological classification of cases was based on the field: it took into consideration the travel history of the patients, date of onset, and entomological data. From the travel history, one out of 54 patients had no travel history to a malaria-endemic country. The transmission of malaria most likely occurred near that patient.

All patients were treated with chloroquine and received a regimen of 14 days of primaquine to kill hypnozoites.

The classification of malaria cases into 1-Imported case, 2-Indigenous case, 3-Introduced case, and 4-Locally acquired case, was performed according to the 2017 WHO definitions [41].

### DNA analysis

Dry blood films from 45 different patients were obtained by scrapping off the surface of the smear with a scalpel and re-suspending in 100 μL of phosphate-buffered saline (PBS) [33]. Genomic DNA was extracted using a QIAamp DNA mini kit (Qiagen) according to the manufacturer’s instructions.

Extracted DNA was first submitted to polymerase chain reaction (PCR) for the plasmodium genus and *Plasmodium vivax* species detection, as previously described [26]. Considering the low amount of DNA collected from these smears, it was not possible to conduct a microsatellite analysis of parasite populations, and genotyping using nested or semi-nested PCR was performed for *Pvcsp*, *Pvmsp1* (fragment 1), and *Pvmsp3 alpha* block II.

All amplification reactions were carried out in a total volume of 20 μL and the presence of 250 nM of each oligonucleotide primer for *Pvcsp* and *Pvmsp1* or 0.1 μM of each oligonucleotide primer for *Pvmsp3 alpha* and 2.0 μL of LightCycler FastStart DNA Master SYBR Green 1 reaction mix.

Primary amplification reactions were initiated with 5.0 μL of the template genomic DNA prepared from Giemsa-stained thin blood smears and 1.0 μL of the product of these reactions was used to initiate the secondary amplification reactions. The cycling parameters for PCR were as follows: an initial denaturation step at 95 °C for 10 min preceded the cycles of annealing at a temperature defined for each primer pair for 2 min for *Pvmsp1* and *Pvcsp* PCRs or 30 s for *Pvmsp3 alpha* PCR, extension step at 72 °C for 2 min for *Pvmsp1* and *Pvcsp* or at 68 °C for 2.5 min for *Pvmsp3 alpha* PCR, and a denaturation step at 95 °C for 1 min for *Pvmsp1* and *Pvcsp* PCRs or 30 s for *Pvmsp3 alpha* PCR.

After a final annealing step followed by 5 min of extension only for *Pvmsp1* and *Pvcsp* PCRs, reaction mixtures from each capillary were collected and stored at 4 °C until secondary PCR or sequencing analysis.

The sequences were determined directly from the PCR-purified templates using a Qiagen DNA purification kit, according to the manufacturer’s instructions. Direct sequencing of the full length of *Pvcsp* and *Pvmsp3 alpha* was performed in both directions using 3730 XL DNA analyzer (Applied Biosystems).

### Sequences analysis

To carry out the study of the potential origin of the samples collected in Oman, nucleotide sequences with the same gene fragment were extracted from GenBank. These reference strains were selected either because they were from eight countries known to be the origin of frequent imported cases in Oman (India, Bangladesh, Pakistan, North Korea, Iran, Mauritania, Brazil, and El Salvador), or because they had the three genes available for the same strain. The accession codes and exact positions of the selected parts of the genes are reported in Table 1.

**Table 1.** GenBank accession numbers and selected sequence position of reference strains.

	Brazil I	India VII	Mauritania I	North Korea	El Salvador I	Iran	Bangladesh	Pakistan
*PvCSP*	KQ234816	KQ234274	RC KQ235043	KQ235379	NC_009913	KT588207		
	507011–507850	433184–433925	1478–2193	59919–60852	1537833–1538631			
*PvMSP1*	KQ234802	KQ234252	KQ235032	KQ235335	NC_009912		AF435620	
	55322–55757	355050–355458	261679–262102	1203–1667	1158314–1158763			
*PvMSP3A*	RC KQ234824	RC KQ234312	RC KQ235063	RC KQ235189	RC NC_009915		AF491951	AY266090
	1212661–1213729	169568–170643	1214086–1215161	256728–257810	1218991–1220078			

Each Omani and reference sequences were trimmed to conserve only the highest quality sequence part, using BioEdit sequence alignment editor, version 7.2.5. The alignments were done through Muscle [9] for *Pvmsp1* and *Pvmsp3 alpha*, and had to be done manually for *csp* due to its repetitive-patterns nature. To calculate the nucleotide diversity of the genes among the Omani sequences compared to the potential diversity of the genes, Oman samples and the reference strains were analyzed using DnaSP, version 5.10.01.

These sequences have been deposited in GenBank under Submission Numbers KY629006–KY6290023 for *pvcsp*, KY629024–KY629045 for *pvmsp1*, and KY629046–KY629070 for *pvmsp3.*

Phylogenetic inferences from concatenated amino acid sequences of Omani samples and reference strains were rooted on *P. cynomolgi* using the maximum likelihood method based on the Tamura-Nei model [36]. *P. cynomolgi* is the closest known relative of *P. vivax*. Initial trees for the heuristic search were obtained automatically by applying neighbor-joining and BioNJ algorithms to a matrix of pairwise distances estimated using the maximum composite likelihood (MCL) approach, and then selecting the topology with the superior log likelihood value [21].

## Results

**Figure 1.**
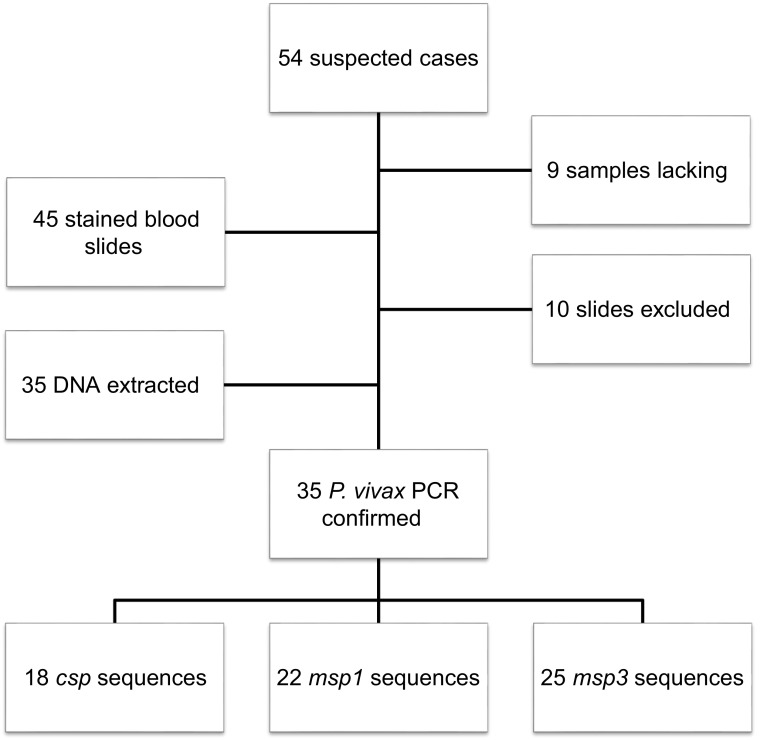
Study flow chart. Ten samples were excluded from the analysis due to lack of identification, DNA degradation, or absence of DNA extracted from scrapping. DNA sequences obtained after PCR for the *Pvcsp*, *Pvmsp1,* and *Pvmsp3 alpha* genes were cured to limit nucleotide errors, to keep sequences of the highest quality.

Fifty-four patients were infected during the outbreak period. The stained slides of nine patients had not been kept. Samples were collected from 45 Giemsa-stained blood smears and tested for plasmodium DNA. Three of these slides were discarded because of identification failure, DNA from five slides was degraded, and we failed to obtain DNA from two slides. Finally, DNA was obtained from 35 slides and submitted to PCR to confirm the presence of *Plasmodium vivax* DNA. These 35 samples were submitted to PCR for the *Pvcsp*, *Pvmsp1*, and *Pvmsp3 alpha* genes, and amplicons were double-stranded sequenced. After manual cleaning of INDEL or sequencing errors, sequences from 18, 22, and 25 samples were selected for *Pvcsp*, *Pvmsp1*, and *Pvmsp3 alpha*, respectively (Figure 1).

The nucleotide diversity (*π*) and the average number of nucleotide differences (*k*) of *Pvcsp*, *Pvmsp1*, and *Pvmsp3 alpha* were *π* = 0.00065, 0.00000, and 0.00100 and *k* = 0.333, 0.00000, and 0.920, respectively. In comparison, those of the reference strains were *π* = 0.04016, 0.143, and 0.02967 and *k* = 9.800, 39.6, and 27.619, respectively.

The circumsporozoite protein gene of *P. vivax,* located on chromosome 8, comprises a central repetitive domain flanked by two conserved domains [17]. The repetitive domain is composed of a 27 bp element repeated a variable number of times. The VK 210 type (type I: GDRADGQPA) and the VK 247 type (type II: ANGAGNQPG) are the most useful markers for *pvcsp* genotyping. We obtained correct *pvcsp* sequencing for 18 samples among the 35. All these 18 samples displayed the VK210 type with a single haplotype (six repeats of GDRADGQPA and nine repeats of GDRAAGQPA).

The merozoite surface protein 1 gene of *P. vivax,* located on chromosome 7, encodes a protein of 190 kDa with 7 interallele conserved blocks and 6 variable blocks. Three segments (F1, F2, F3) have been described for *P. vivax* genotyping [31]. The F1 fragment, located at variable block 2, was sequenced here. Twenty-two samples provided good quality sequences and were further analyzed. Only one haplotype could be detected using five short tandem repeats (tripeptides) (Table 2), compared to the different haplotypes of the reference sequences from seven different areas of transmission.

**Table 2.** Comparison of peptide repeat motifs (PRM) from seven countries to those of samples from Oman for *csp*, *msp1*, and *msp3 alpha*. Numbers highlighted in bold are samples with genotyping of three markers.

Sample ID	*csp*		*msp1*					*msp3 alpha*	
	VK210	VK210	SSE	SSG	SSV	GSS	GTG	KKAE	KKAK
	A	B							
Brazil	6	4	1	0	0	2	1	1	1
El Salvador	10	6	1	0	0	3	1	1	1
Mauritania	4	3	1	1	2	1	1	1	1
Iran	7	8	–	–	–	–	–	–	–
India	4	4	0	0	0	0	0	1	1
Bangladesh	–	–	1	0	0	1	1	1	1
North Korea	3	4	1	0	0	1	1	1	1
**2**	**6**	**10**	**1**	**1**	**2**	**1**	**1**	**1**	**1**
3								1	1
4			1	1	2	1	1		
6								1	1
7			1	1	2	1	1		
8			1	1	2	1	1	1	1
9			1	1	2	1	1		
12	6	10						1	1
13								1	1
17								1	1
19			1	1	2	1	1	1	1
21	6	10						1	1
22			1	1	2	1	1	1	1
23			1	1	2	1	1	1	1
**24**	**6**	**10**	**1**	**1**	**2**	**1**	**1**	**1**	**1**
25			1	1	2	1	1	1	1
**26**	**6**	**10**	**1**	**1**	**2**	**1**	**1**	**1**	**1**
27			1	1	2	1	1		
28			1	1	2	1	1		
31			1	1	2	1	1		
32			1	1	2	1	1	1	1
33			1	1	2	1	1		
34			1	1	2	1	1		
35								1	1
36			1	1	2	1	1	1	1
37	6	10						1	1
38	6	10							
39	6	10	1	1	2	1	1		
40	6	10							
41								1	1
**42**	**6**	**10**	**1**	**1**	**2**	**1**	**1**	**1**	**1**
43	6	10						1	1
45	6	10						1	1
46	6	10						1	1
47	6	10						1	1
48	6	10	1	1	2	1	1		
51	6	10							
52	6	10						1	1
53	6	10	1	1	2	1	1		

The merozoite surface protein 3 alpha of *P. vivax* is a member of the *msp3* family (*α*, *β*, *γ*), located on chromosome 10, which encodes a protein with a predominant central alanine-rich domain [18]. This gene has been extensively used because of its high genetic diversity. Two blocks displayed in *Pvmsp3 alpha* have been studied, block I (residues 104–396) and block II (434–687), which is known to be relatively well conserved. Its main non-random polymorphisms appear in motif I from amino acid positions 533 to 538 and in motif II from 580 to 587 [13, 27, 29]. Our analysis, focused on residues 378–688, enabled us to analyze the block II motifs. The strains from Oman, Pakistan, and El Salvador displayed the motif association MSELEK/TANVVKD, those from North Korea, Mauritania, and India displayed MSELEK/KEATAAKL, the strain from Brazil displayed LSKLEE/TAANVVKD, and the strain from Bangladesh displayed LSKLEE/KEATAAKL. While the Omani samples all displayed the same motifs, two of them had an error-like non-sense mutation. Short tandem repeats were detected in all the sequences, but the haplotypes were similar in all the Omani samples and in the reference sequences, and no information could be obtained from these sequence analyses.

**Figure 2.**
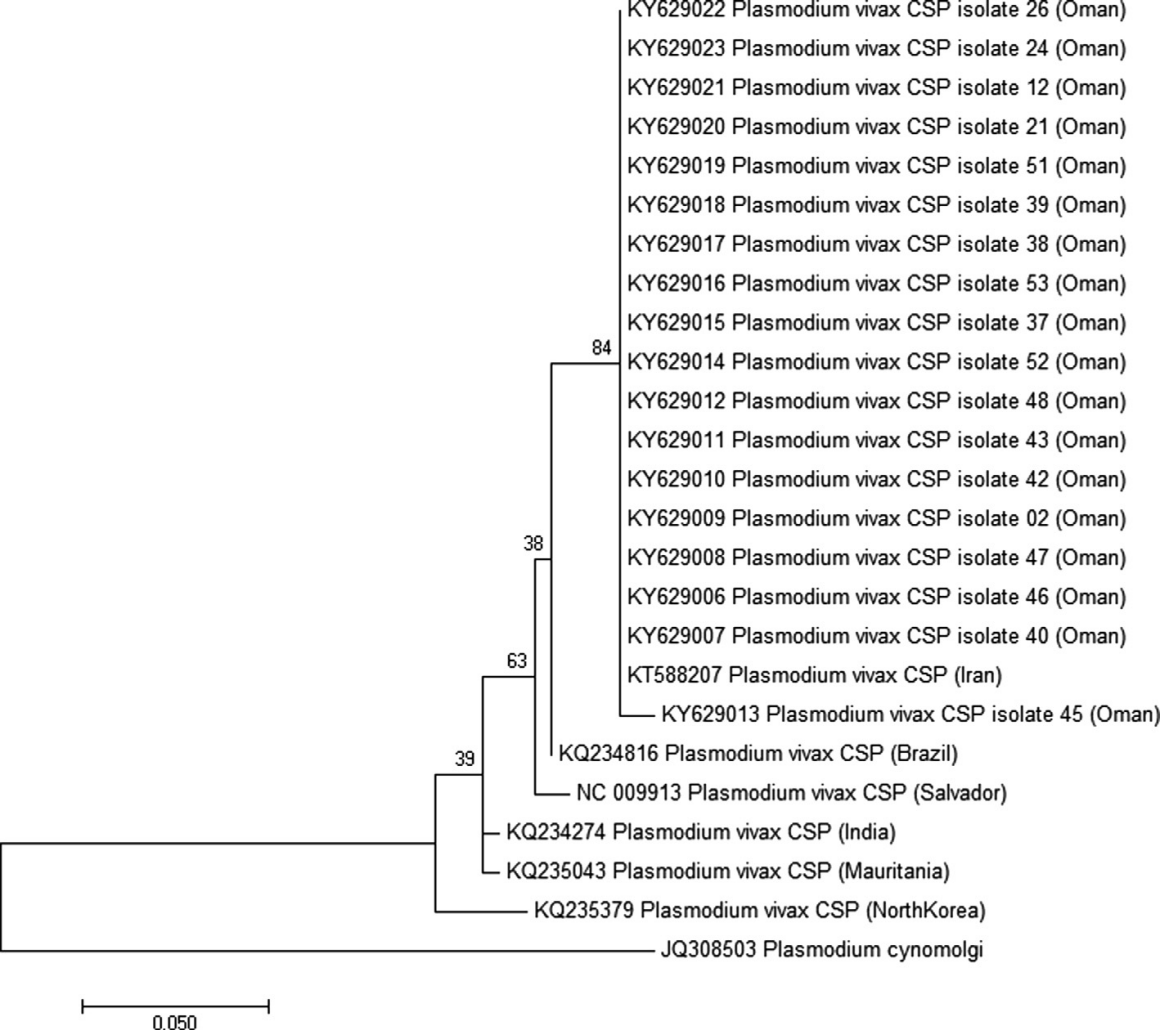
Molecular phylogenetic analysis by maximum likelihood method for the 18 sequences of *Pvcsp* protein from Oman and six reference strains. The evolutionary history was inferred by using the *maximum likelihood method* based on the Tamura-Nei model [36]. The tree with the highest log likelihood (−587.1315) is shown. The tree is drawn to scale, with branch lengths measured in the number of substitutions per site. The analysis involved 25 nucleotide sequences. All positions containing gaps and missing data were eliminated. There were a total of 220 positions in the final dataset. Evolutionary analyses were conducted in MEGA7 [21]. The tree was rooted on *Plasmodium cynomolgi csp*. Bootstrap test results are shown next to the branches.

**Figure 3.**
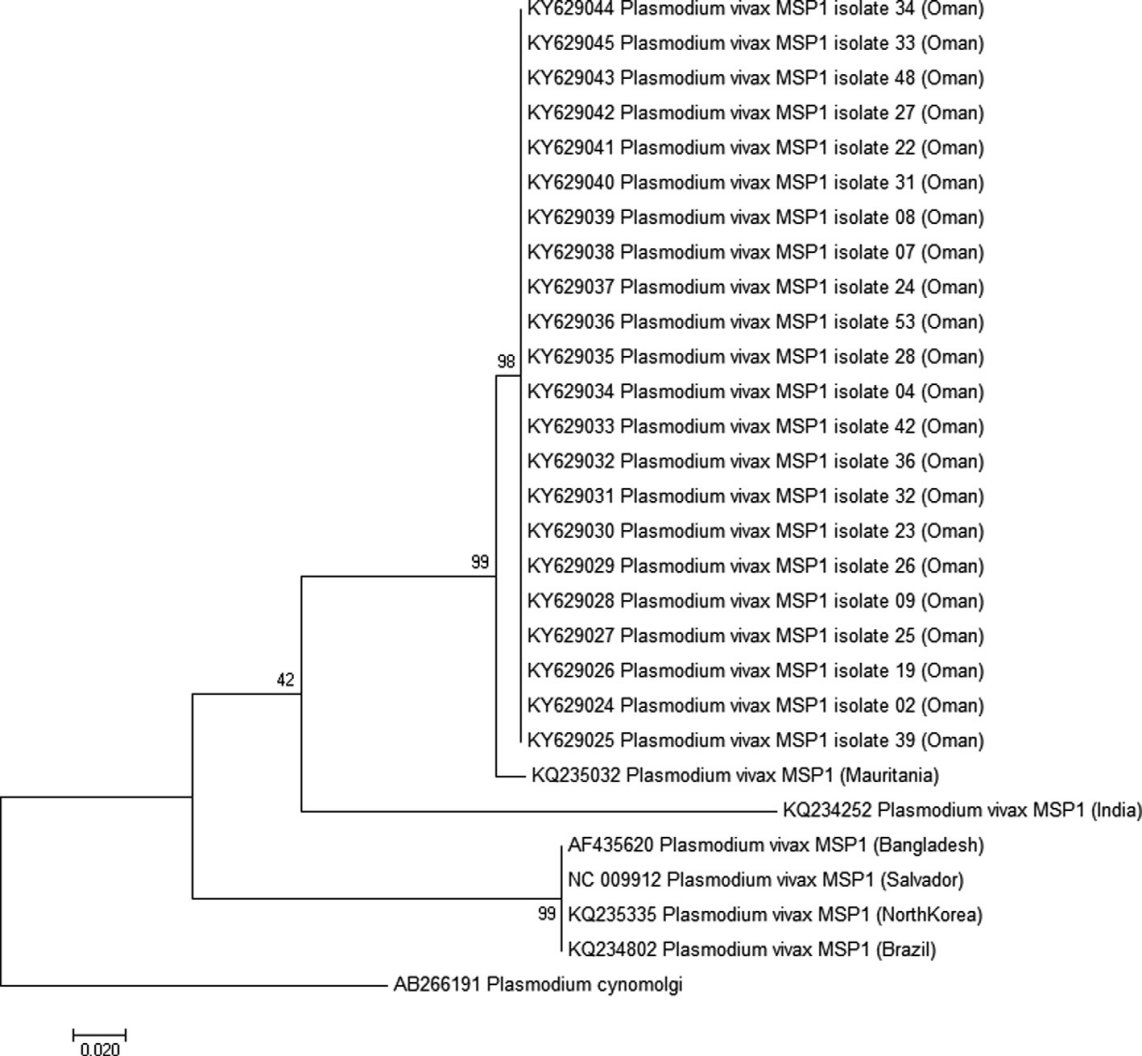
Molecular phylogenetic analysis by maximum likelihood method for the 22 sequences of *Pvmsp1* protein from Oman and six reference strains. The evolutionary history was inferred by using the maximum likelihood method based on the Tamura-Nei model [36]. The tree with the highest log likelihood (−880.6611) is shown. The tree is drawn to scale, with branch lengths measured in the number of substitutions per site. The analysis involved 29 nucleotide sequences. All positions containing gaps and missing data were eliminated. There were a total of 243 positions in the final dataset. Evolutionary analyses were conducted in MEGA7 [21]. The tree was rooted on *Plasmodium cynomolgi msp1*. Bootstrap test results are shown next to the branches.

**Figure 4.**
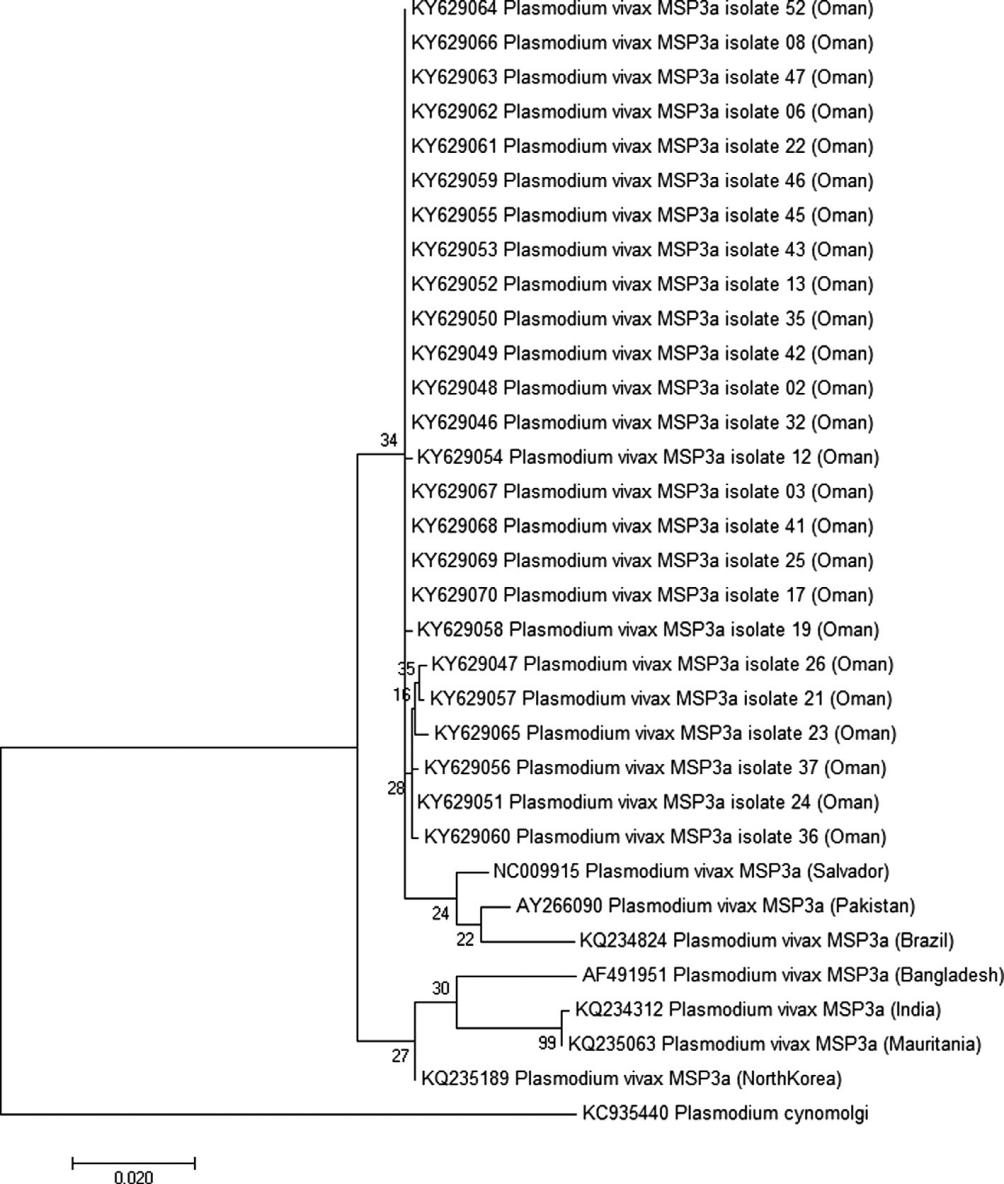
Molecular phylogenetic analysis by maximum likelihood method for the 25 sequences of *Pvmsp3 alpha* protein from Oman and seven reference strains. The evolutionary history was inferred by using the maximum likelihood method based on the Tamura-Nei model [36]. The tree with the highest log likelihood (−2208.3432) is shown. The tree is drawn to scale, with branch lengths measured in the number of substitutions per site. The analysis involved 33 nucleotide sequences. All positions containing gaps and missing data were eliminated. There were a total of 895 positions in the final dataset. Evolutionary analyses were conducted in MEGA7 [21]. The tree was rooted on *Plasmodium cynomolgi msp3a*. Bootstrap test results are shown next to the branches.

Phylogenetic inference showed that Omani samples did not share a node with most of the reference strains (Figures 2–4). Surprisingly, samples from Oman were not closely related to samples from India, Bangladesh, or Pakistan, while most of the patients included came from these countries. Using *Pvcsp*, the reference sequence from Iran showed the lowest divergence with the Omani samples (Figure 2). Unfortunately, no *Pvmsp1* and *Pvmsp3 alpha* sequences from Iran were available from GenBank to confirm this relationship (Figures 3 and 4).

## Discussion

Oman has managed to control local transmission of malaria, and for the first time no local transmission of malaria was reported in 2004. This is of the utmost importance for Oman, since potential re-introduction of the parasite would have social, economic, and tourism consequences. The Omani population is divided into Omani nationals (approx. 54% of the population) and migrant workers (approx. 46% of the population). One of the major concerns for the Omani Health Authorities is that migrants mainly originate from countries in Southeast Asia with endemic *P. vivax* malaria. These immigrants work for several months on building construction sites and in agriculture, live in close proximity to water tanks, and are thus subject to mosquito bites.

An outbreak of malaria cases was detected by staff at the Department of Malaria, Ministry of Health, Oman, and stained blood smears were obtained from finger pricks and used for the diagnosis. Venous blood samples were not collected at the time of diagnosis and patients were not followed up. Thus, the only DNA material available to investigate the outbreak was the stained thick blood smears. The low quality and quantity of this material preclude a deep genetic analysis and it was not possible to use microsatellite analysis or whole-genome sequencing. DNA was obtained from 35 slides, and PCR sequencing made it possible to obtain 18, 22, and 29 sequences of the conventional markers *Pvcsp*, *Pvmsp1*, and *Pvmsp3 alpha*, respectively.

The measures of genetic polymorphism using nucleotide diversity (*π*) and average number of nucleotide differences (*k*) showed that all 35 samples were very closely related and thus most probably originated from a single index case. This is in agreement with the hypothesis of a series of secondary locally acquired cases contracted from a gametocyte carrier infected outside Oman. These carriers may have been in the pre-patency or patency period if recently infected, or in a long-term patency period if infected with a temperate parasite genotype.

To identify the genotype of this *P. vivax* parasite population in Oman, genotyping was done using the *Pvcsp* gene. All the isolates showed the VK210 pure genotype, demonstrating a temperate genotype leading to the possibility of long latency between infection and reviviscence. Thus, it could be hypothesized that the cases diagnosed during this outbreak were the primary attacks of *P. vivax* malaria transmitted from a limited number or single case of symptomatic or asymptomatic carriers previously infected in a country where temperate genotypes are circulating. It should be noted that the VK210 genotype is also predominant in countries from which many Omani migrants originated, including Pakistan, Afghanistan [42, 43], and India [19]. These results may also indicate that among the species of Anopheline vectors susceptible to transmit VK210 parasites, *A. culicifacies*, detected in water tanks around the cases, is most probably the vector [35].

The nearly identical *Pvmsp3 alpha* genotype of the Omani parasites demonstrates a common origin of the cases. In fact, other studies analyzing isolates from different regions of a country displayed more polymorphism in this gene than in the collection of Omani parasites [7, 13, 37]. Motifs I and II were the same for all the Omani parasites. Two reference strains from Pakistan and El Salvador also showed this combination, but the North Korean strain which showed the highest global phylogenetic proximity to Oman parasites had a different motif.

None of the markers used allowed us to definitively establish the origin of the outbreak. Considering that the highest diversity will be observed in an area with high transmission, it could be considered that the divergence of the parasites involved in this outbreak compared to the reference sequences from Asian countries could lead us to suspect a different origin of the parasites introduced in Oman.

The proximity of *Pvcsp* sequences observed in Oman with a sequence from Iran deposited in GenBank (2016) does not lead to the definitive conclusion that the Omani cases were imported from Iran. The lack of available sequences of *Pvmsp1* and *Pvmsp3 alpha* from Iran precludes more detailed analysis. Migrant workers from Iran are numerous in Oman, but most of them are not exposed to the poor environmental conditions that the freelance construction workers from the Indian subcontinent are subjected to. There were no Iranian migrant workers in the area where the malaria outbreak occurred. Samples collected from Iran should be compared to Omani sequences to confirm this hypothesis.

Alternatively, the hypothesis of an Omani *P. vivax* circulating in the country and causing small outbreaks like this one is highly unlikely, given that no new cases have been seen since the outbreak ended.

## Conflict of interest

The Associate Editor of Parasite, Stéphane Picot, is one of the authors of this manuscript. COPE (Committee on Publication Ethics, http://publicationethics.org), to which Parasite adheres, advises special treatment in these cases. In this case, the peer-review process was handled by the Editor-in-Chief.
